# Characteristics of the fecal microbiome and metabolome in older patients with heart failure and sarcopenia

**DOI:** 10.3389/fcimb.2023.1127041

**Published:** 2023-02-24

**Authors:** Jieting Peng, Hui Gong, Xing Lyu, Yang Liu, Shizhen Li, Shengyu Tan, Lini Dong, Xiangyu Zhang

**Affiliations:** ^1^ Department of Geriatrics, The Second Xiangya Hospital, Central South University, Changsha, Hunan, China; ^2^ Laboratory of Clinical Medicine, The Second Xiangya Hospital, Central South University, Changsha, Hunan, China

**Keywords:** sarcopenia, heart failure, gut microbiota, short-chain fatty acids, 16S rRNA sequencing

## Abstract

**Background:**

Increasing evidence supports that gut microbiota plays an important role in the development of cardiovascular diseases. The prevalence of sarcopenia is increasing in patients with heart failure. Muscle wasting is an independent predictor of death in heart failure patients.

**Aims:**

In this study, we aimed to explore the characteristics of gut microbiota and metabolites in heart failure patients with or without sarcopenia.

**Methods:**

Fecal samples of 33 heart failure patients without sarcopenia, 29 heart failure patients with sarcopenia, and 15 controls were collected. The intestinal microbiota was analyzed using 16S rRNA sequencing and the metabolites were detected using the gas chromatography-mass spectrometry method.

**Results:**

There were significant differences in the overall microbial community structure and diversity between control and heart failure patients with or without sarcopenia. However, no clear clustering of samples was observed in heart failure with and without sarcopenia patients. Several bacterial, particularly *Nocardiaceae*, *Pseudonocardiaceae, Alphaproteobacteria*, and *Slackia* were significantly enriched in the heart failure patients without sarcopenia, while *Synergistetes* was more abundant in the heart failure patients with sarcopenia. Isobutyric acid, isovaleric acid, and valeric acid were lower in heart failure patients with sarcopenia than that without sarcopenia but lacked significance.

**Conclusions:**

This study demonstrates that there are differences in the gut microbiota between control individuals and heart failure patients with or without sarcopenia. Modulating the gut microbiota may be a new target for the prevention and treatment of sarcopenia in heart failure patients.

## 1 Introduction

Along with population aging, geriatric syndromes have become a major healthcare problem worldwide. Sarcopenia is one of the most challenging geriatric syndromes with multiple contributing factors. It has been described as an age-related decline in skeletal muscle mass as well as muscle function, which could lead to a decrease in physical capability, falls, disability, and higher mortality in older people (Cruz-Jentoft et al., 2010). On the other hand, chronic heart failure (CHF) remains one of the most important healthcare problems, with high costs and poor outcomes. The prevalence of heart failure (HF) is more than 10% among people aged ≥70 years. Sarcopenia is a common complication of CHF patients and the prevalence of sarcopenia in CHF patients was nearly 20% (Fulster et al., 2013). The prognosis of HF with sarcopenia is worse than that of patients without sarcopenia (Narumi et al., 2015). A prospective cohort study revealed that sarcopenia was significantly linked with a cardiac event in the New York Heart Association Class II–IV group (Zhang et al., 2021). In addition, a multicenter prospective cohort study indicated that sarcopenia was an independent predictor of 1-year mortality in both ejection fraction preserved and reduced HF patients (Konishi et al., 2021).

More than 1000 different bacterial species are observed in the gut. The diversity of the bacterial species plays a major role in maintaining homeostasis (Przewlocka et al., 2020). The composition of gut microbiota is proven to be dynamic due to dietary changes, age, disease, and so on (Marti et al., 2017). The gut microbiome is being increasingly recognized as a modulator of atherosclerosis, hypertension, atrial fibrillation, and myocardial fibrosis and contributes to the development of these diseases (Peng et al., 2018). Accumulating evidences suggest that gut microbiota has a significant impact on skeletal muscle metabolism and is related to the development of HF (Branchereau et al., 2019; Lahiri et al., 2019). Gut microbiota-derived micronutrients and metabolites such as lipopolysaccharide, trimethylamine N-oxide, short-chain fatty acids (SCFAs), and secondary bile acids can reach and act on muscle (Liu et al., 2021). Gut microbiome affects HF by generating bioactive metabolites that can impact host physiology (Tang et al., 2019). Numerous studies have demonstrated the existence of the gut microbiome-muscle axis and heart-gut microbiome axis (Liao et al., 2020; Madan and Mehra, 2020). Germ-free mice had less skeletal muscle mass than pathogen-free mice when fed the same food (Lahiri et al., 2019). Besides, gut microbiota influenced HF mainly by affecting immunity and inflammation (Yuzefpolskaya et al., 2020). Modulating the microbiota through dietary intervention, probiotics or prebiotics supplements, may be a novel strategy against muscle aging and HF.

SCFAs including acetate, propionate, butyrate, isovalerate, valerate, and caproate, are produced by microbial fermentation of fiber and prebiotics in the colon and mediate the interaction among diet, microbiota, and the host (Kimura et al., 2014). A study found that treating germ-free mice with SCFAs partially reversed muscle atrophy induced by dexamethasone (Lahiri et al., 2019). Professional athletes had relatively increased SCFAs compared with more sedentary subjects (Barton et al., 2018). SCFAs influence HF by regulating T-cell proliferation and exerting anti-inflammatory effects (Mamic et al., 2021) and proved to be an efficient energy source during pathological stress in the failing heart (Carley et al., 2021). Meanwhile, SCFAs were reported to have a positive impact on reducing cardiac hypertrophy and pressure overload (Pakhomov and A. Baugh, 2021).

Emerging evidences have shown the association between gut microbiota and sarcopenia. Sarcopenia may accelerate the progression of HF and increase the mortality of patients with HF. However, there has no standard treatment for slowing muscle loss in patients with HF. It is not clear whether alterations in gut microbiota and metabolites are related to sarcopenia in HF patients. This study is the first to explore the changes of gut microbiota composition and SCFAs levels in HF patients with or without sarcopenia.

## 2 Methods

### 2.1 Study participants

Seventy-seven patients aged ≥ 65 years old with similar diet and environmental conditions in the Second Xiangya Hospital of Central South University were enrolled in this study. Patients were classified into the following 3 categories: 33 HF patients without sarcopenia (HF group), 29 HF patients with sarcopenia (SHF group), and 15 control individuals (Control group). Sarcopenia was diagnosed according to the Asian Working Group for Sarcopenia 2019 Guidelines (Chen et al., 2020). Low skeletal muscle mass was defined as muscle mass < 7.0 kg/m^2^ (male) or < 5.7 kg/m^2^ (female) by bioelectrical impedance analysis using the InBodyS10 body composition analyzer (Chen et al., 2014). Low muscle strength was defined as handgrip strength <28 kg for male and <18 kg for female. Criteria for low physical performance is a 6-m walk speed < 1 m/s. Sarcopenia was defined as low muscle mass plus either diminished muscle strength or physical performance. Exclude subjects included recurrent diarrhea or constipation, unusual dietary habits (vegetarians), edema, those with tumors, diabetes, intestinal inflammation, irritable bowel syndrome, history of intestinal surgery, being treated with antibiotics or probiotics within 1 month. Demographic characteristics and clinical laboratory examinations were documented for all patients. The study was approved by the local Ethics Committee of the Second Xiangya Hospital of Central South University. Written informed consent was obtained from all participants. This study was conducted under the Declaration of Helsinki.

### 2.2 Fecal samples collection

Fecal samples of the patients were collected within one hour of excretion in the morning during hospitalization and stored at -80°C in preservation tubes before being delivered to the detected center.

### 2.3 16S rRNA gene sequencing

Microbial genomic DNA of fecal samples was extracted by the Quant-iT PicoGreen dsDNA Assay Kit (Invitrogen) according to the manufacturer’s instructions. The purity and integrity of total DNA were detected using the NanoDrop NC-2000 spectrophotometer (Thermo Fisher Scientific, United States) and 1.2% agarose gel electrophoresis. Hypervariable regions V3–V4 of the 16S rRNA gene were amplified by polymerase chain reaction (PCR) using the primer ACTCCTACGGGAGGCAGCA and GGACTACHVGGGTWTCTAAT and sequenced by the NovaSeqPE250 according to the manufacturer’s manuals.

### 2.4 Fecal short-chain fatty acid analysis

SCFAs quantification was evaluated by the gas chromatography-mass spectrometry method (GC-MS). An appropriate stool sample was taken and 50 μL 15% phosphoric acid, 100 μL 125 μg/mL isocaproic acid, and 400 μL ether were added; then placed in a high-throughput tissue grinder and grind at 55 Hz for 60 s twice. The samples were centrifuged and the supernatant was collected. The mixture was briefly vortexed before GC-MS analysis. A Thermo TRACE, 1310-ISQ LT GC-MS system (Thermo, USA) was used to perform the analysis.

### 2.5 Bioinformatics analysis

The DADA2 plugin is used for quality filtering, denoising, merging and removing chimeras of sequences. Data were filtered by removing low-quality reads. Briefly, the entire sequence was removed if the average quality < 20 and the read length after truncation was 75% lower than the original read length. Primer/Adaptor- containing reads, N-containing reads were also removed to obtain high-quality clean data. After quality control, the raw data were filed, and the optimized sequences were identified, and then UPARSE (version7.1) were used to assign the raw sequences to operational taxonomic units (OTUs) based on OTUs having≥97% similarity. Each OTU is considered to represent each taxonomic level, i.e., kingdom, phylum, class, order, family, and genus. By comparing the RDP Classifier algorithm (http://rdp.cme.msu.edu/) with the Silva (SSU123) 16S rRNA database, the taxonomy of each 16S rRNA gene sequence was analyzed based on a 70% confidence threshold. Bioinformatics analyses were conducted using QIIME (version 1.9.1). The diversity within the gut community was assessed by alpha diversity and beta diversity. Alpha diversity indices include the Chao1 richness estimator, Shannon, and Inverse Simpson (Chen et al., 2021). Beta diversity includes principal coordinate analysis (PCoA) plots and nonmetric multidimensional scaling (NMDS) which were created to visualize the structural variation of microbial communities between different groups. Additionally, to further detect differentially abundant taxa in the community structure (phylum and genus) between the groups of samples, the linear discriminant analysis effect size (LEfSe) method was used to compare the differences in the taxonomic levels.

### 2.6 Statistical analyses

Statistical analyses were performed using SPSS 25.0 software and R (3.0.2). Normal distribution data, non-normal distribution data and categorical data were expressed as mean ± standard deviation (SD), median with interquartile range and number (%) respectively. Normal distribution data were analyzed using Student’s t-test between two groups, non-normal distribution data were analyzed using Mann Whitney U-test. Statistical analysis of beta diversity among different groups was conducted using the adonis test. Kruskal-Wallis tests were applied to determine the statistical differences in the relative abundance of OTUs and alpha diversity indexes between different groups (*P*< 0.05). Correlations between SCFAs and different microbiota were evaluated using Spearman rank correlation, and presented as a heatmap.

## 3 Results

### 3.1 Demographic and clinical characteristics of the subjects

Clinical characteristics including sex, age, history of smoking and drinking, blood lipid parameters, and history of the disease and medication were obtained from medical records. Body mass index (BMI) in the SHF group was significantly lower than that in the control and HF groups, while ALT and Cr were significantly higher in the HF group than that in the control group. The percentages of diuretic and angiotensin-converting enzyme inhibitor/angiotensin receptor blocker use in the control group were significantly lower than that in the HF and SHF groups. There were no significant differences in sex, age, WBC, RBC, AST, percentages of smoking and drinking, percentages of β-receptor blocker, statin, and calcium channel blocker use among the three groups (**Table 1**).

**Table 1:** The demographic and clinical characteristics of the subjects.

	HF (n=33)	SHF (n=29)	Control (n=15)	*P*
Sex (Male, %)	24 (72.70%)	13 (44.80%)	8 (53.30%)	0.076
Age (years)	71.76 ± 7.93	75.14 ± 8.18	67.67 ± 9.76	0.059
BMI (kg/m^2^)	24.24 ± 2.81^###^	20.27 ± 3.75	23.52 ± 3.12^#^	0.000
WBC (×10 ^9^/L)	5.56 (4.83, 6.29)	5.16 (4.35, 7.25)	4.90 (4.14, 5.99)	0.437
RBC (×10 ^9^/L)	4.05 ± 0.84	4.00 ± 0.54	4.28 ± 0.42	0.300
ALT (IU/L)	19.80 (16.90, 23.30)^*^	15.10 (11.00, 30.70)	13.10 (11.10, 15.60)	0.002
AST (IU/L)	17.60 (11.80, 23.20)	20.90 (14.90, 36.00)	15.70 (14.30, 17.65)	0.055
Cr (μmol/L)	97.00 (77.20, 112.30)^*^	83.00 (63.30, 122.50)	73.00 (58.00, 89.00)	0.018
Smoking, n (%)	14 (42.40%)	11 (37.90%)	5 (33.30%)	0.827
Drinking, n (%)	11 (32.30%)	9 (31.00%)	2 (13.30%)	0.340
Diuretic, n (%)	20 (60.60%)	11 (37.90%)	NA	NA
ACEI/ARB, n (%)	25 (75.80%)^***^	22 (75.90%)^***^	2 (13.30%)	0.000
β-receptor blocker, n (%)	23 (69.70%)	25 (86.20%)	8 (46.70%)	0.059
Statin, n (%)	27 (81.80%)	25 (86.20%)	11 (73.30%)	0.577
CCB, n (%)	6 (18.18%)	9 (31.03%)	5 (33.33%)	0.396

BMI, body mass index; WBC, white blood cell count; RBC, red blood cell count; ALT, alanine transaminase; AST, aspartate transaminase; Cr, creatinine. ACEI/ARB, angiotensin-converting enzyme inhibitor/angiotensin receptor blocker. CCB, calcium channel blocker. NA, not available. *p < 0.05, ***p < 0.001 compared to control group. ^#^p ;< 0.05, ^###^p < 0.001 compared to SHF group.

The body composition parameters and echocardiographic data of the three groups were compared. There were no significant differences among the control, HF, and SHF groups in the visceral adipose area, body fat, left ventricular end-diastolic diameter (LVEDd), right atrial diameter (RAD), and right ventricular end-diastolic diameter (RVEDd). The HF and SHF groups had significantly higher N-terminal pro-brain natriuretic peptide (NT-proBNP) levels, left atrial diameter (LAD), and lower left ventricular ejection fraction (LVEF) compared to the control group (**Table 2**). Basic metabolic rate, bone mineral content, upper arm circumference, and SMI were significantly lower in the SHF group when compared to the control and HF groups (*p*<0.05) (**Table 2**). The results are consistent with the characteristics of HF and sarcopenia patients.

**Table 2:** Body composition and cardiac parameters of the subjects.

	HF (n=33)	SHF (n=29)	Control (n=15)	*P*
Visceral adipose area	84.00 (68.55, 102.80)	67.30 (55.05, 93.25)	70.10(51.60, 89.95)	0.096
Basic metabolic rate	1397.00 (1268.00, 1515.00) ^###^	1151.00 (1073.00, 1298.50)	1280.00 (1230.00, 1582.00) ^##^	0.004
Body fat (%)	26.33 ± 7.91	25.61 ± 9.78	25.11 ± 6.85	0.758
Bone mineral content	2.65 ± 0.35^###^	2.29 ± 0.40	2.66 ± 0.46^#^	0.000
Upper arm circumference	30.13 ± 2.98^###^	26.47 ± 2.49	29.83 ± 2.96^##^	0.000
SMI	7.42 ± 0.93^###^	5.73 ± 0.77	7.14 ± 1.02^##^	0.000
NT-pro BNP	1084.00 (372.00,3200.00)^***^	1424.00 (514.00, 4830.00)^***^	73.60 (27.10, 120.00)	0.000
LVEDd	49.76 ± 7.72	47.66 ± 8.50	44.87 ± 3.52	0.123
LAD	39.27 ± 7.25^**^	39.14 ± 6.47^**^	31.73 ± 6.81	0.001
RVEDd	30.30 ± 3.44	30.48 ± 4.56	28.13 ± 1.51	0.073
RAD	30.00 (28.00, 36.00)	31.00 (26.50, 36.50)	28.00 (27.00, 29.50)	0.081
LVEF (%)	57.00 (39.50, 61.50)^*^	55.00 (38.00, 60.00)^*^	63.00 (60.00, 65.00)	0.010

SMI, skeletal muscle index; NT-pro BNP, N-terminal pro-brain natriuretic peptide; LVEDd, left ventricular end-diastolic diameter; LAD, left atrial diameter; RAD, right atrial diameter; RVEDd, right ventricular end-diastolic diameter; LVEF, left ventricular ejection fraction. *p < 0.05, **p < 0.01, ***p < 0.001 compared to control group. ^#^p < 0.05, ^##^p < 0.01, ^###^p < 0.001 compared to SHF group.

### 3.2 Alpha and beta diversity of gut microbiota

**Figure 1:**
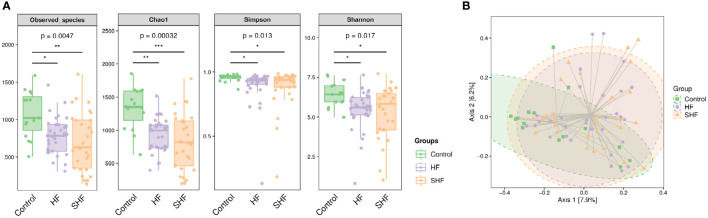
Gut microbial diversity in three groups. **(A)** Alpha diversity index measured by Observed, Chao1, Shannon, and Simpson methods. The boxplots are showing interquartile (IQR) ranges with the median and whiskers extending up to the most extreme point within 1.5 folds IQR. Figures under the diversity index label are p-values from the Kruskal-Wallis test. **(B)** Beta diversity index measured by Bray-Curtis method using PCoA based on OTUs relative abundance profile. The two-variance explained by Axis.1 and Axis.2 are 7.9% and 6.2%, respectively. *p <0 .05, **p < 0.01, ***p < 0.001 compared to control group.

The gut microbial composition was compared in three groups. There were significant differences among the control, HF group, and SHF group in diversity (Observed-species index and Chao1) and abundance (Simpson and Shannon indexes) (**Figure 1A**). These indexes were similar between the HF group and the SHF group. Beta diversity reflects the between-habitat diversity in microbial community structure assessed using Bray and Curtis distances. Axis.1 and Axis.2 explained 7.9% and 6.2% of the variation in microbiota, respectively. Significant separations were found between the control group with HF or SHF group (*p* = 0.002). No significant differences were observed in microbial community composition between HF and SHF group (*p* = 0.641) (**Figure 1B**). These findings suggested gut microbial dysbiosis in HF and SHF groups compared to the control group.

### 3.3 Gut microbiota composition at phylum and genus levels

**Figure 2:**
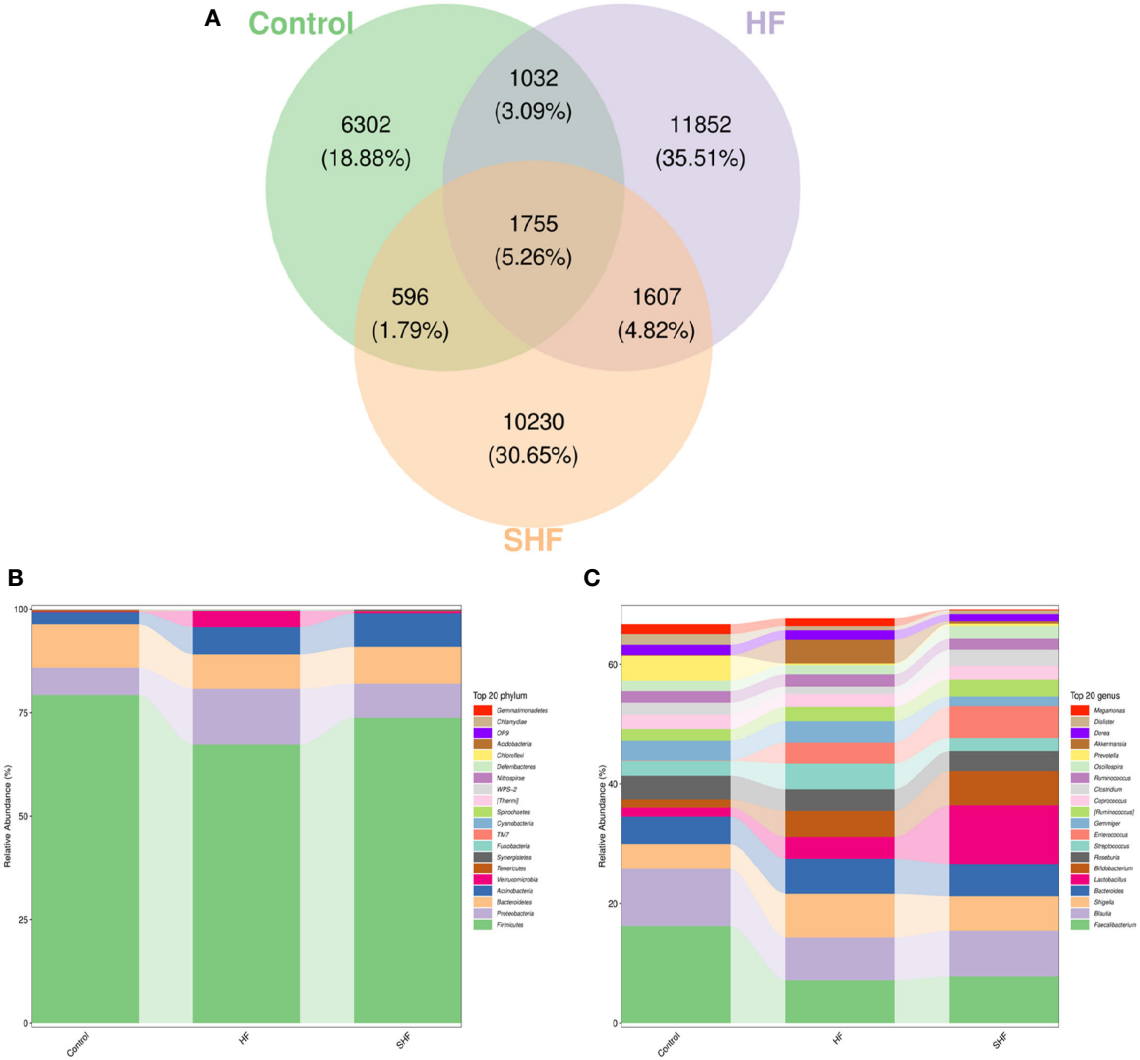
Gut microbiota compositional shifts at phylum and genus level. **(A)** Venn diagram of OTUs shared by and exclusive to the three groups. Each ellipse represents a group, the overlapping area between the ellipses indicates the shared OTU between the groups and the number indicates the number of OTUs. Corresponding percentages are noted for relevant overlaps. The overall bacterial structures of the three groups at **(B)** phylum and **(C)** genus levels are expressed as the relative abundance of OTUs in each group (The top 20 phylum and genus with the average OTU frequency of the sample).

As shown in **Figure 2A**, each ellipse represents a group, the overlapping area between the ellipses indicates the shared OTU between the groups and the number indicates the number of OTUs. There were, 1755 shared OTUs in the three groups, while 6302, 11852, and, 10230 OTUs were unique to the control, HF, and SHF groups respectively. Similar to previous studies, the microbial communities in the human gut mainly belong to *Firmicutes*, *Bacteroidetes*, *Proteobacteria*, *Actinobacteria*, and *Verrucomicrobia* (**Figure 2B**). At the phylum level, *Firmicutes* and *Bacteroidetes* were more abundant in the control group than in the HF and SHF group. In the HF and SHF groups, the relative abundance of *Proteobacteria* and *Actinobacteria* was higher than that in the control group (**Figure 2B**). At the genus level, *Faecalibacterium*, *Blautia*, and *Prevotella* were more abundant in the control group than in the HF and SHF groups (**Figure 2C**). Compared with the SHF group, the HF group displayed a higher abundance of *Shigella*, *Bacteroides*, *Streptococcus*, *Gemmiger*, and *Akkermansia*, while lesser in *Lactobacillus*, *Bifidobacterium*, *Enterococcus* (**Figure 2C**).

### 3.4 Different abundance of gut microbiota in the three groups

**Figure 3:**
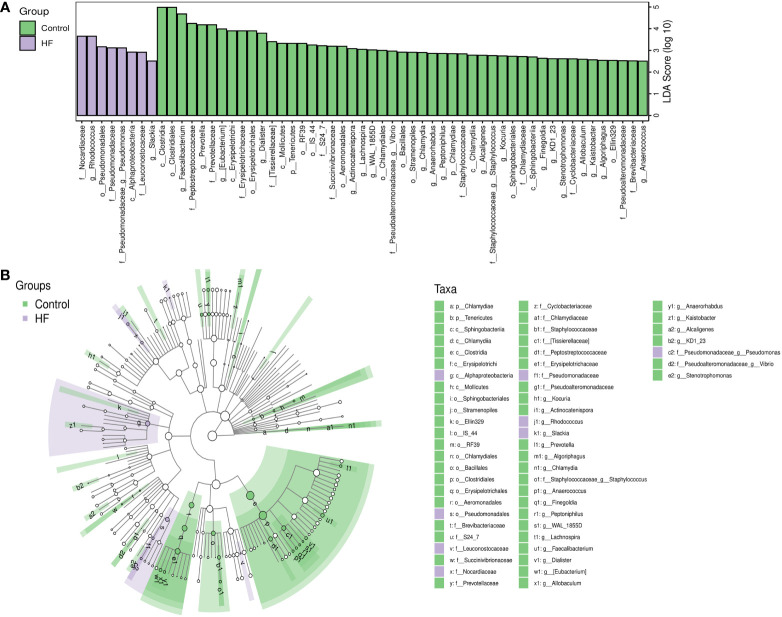
The different abundance of gut microbiota between Control and HF group. Gut microbial markers were measured by LEfSe analysis (cut-off value LDA > 2.5) between the Control and HF group. Histograms **(A)**; Cladogram **(B)**.

**Figure 4:**
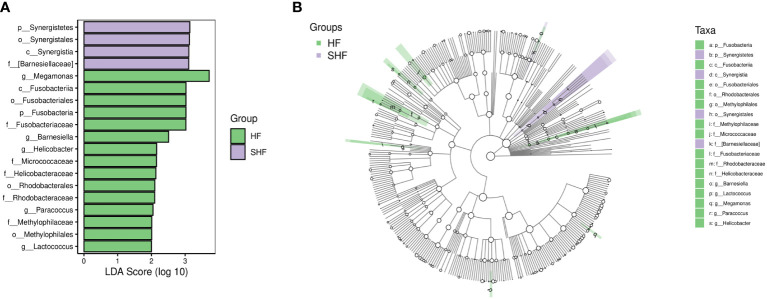
The different abundance of gut microbiota between the HF and SHF group. Gut microbial markers were measured by LEfSe analysis (cut-off value LDA > 2.0) between the HF and SHF group. Histograms **(A)**; Cladogram **(B)**.

LEfSe analysis based on the Linear Discriminant Analysis (LDA) score was used to measure the unique microbial characteristics that distinguished each group. The results showed that the *Nocardiaceae*, *Pseudonocardiaceae*, *Leuconostocaceae*, and *Alphaproteobacteria*, *Slackia* were significantly more enriched in the HF group when compared to the control group (**Figures 3A, B**). Meanwhile, the *Synergistetes* was significantly enriched in the SHF versus the HF group (**Figures 4A, B**). Furthermore, compared with the HF and SHF groups, the control group had a higher abundance of *Clostridia*, *Faecalibacterium, Peptostreptococcaceae*, and *Prevotellaceae* (**Figures 3A, B**).

### 3.5 Fecal levels of SCFAs in Control, HF, and SHF groups

The concentrations of SCFAs were assessed in feces. Fecal levels of acetic acid, propionate acid, butyric acid, and caproic acid were similar in the three groups (**Table 3**). The levels of isobutyric acid, isovaleric acid, and valeric acid were lower in the SHF group compared with that in the HF and control group but has no significant differences (**Table 3**).

**Table 3:** Fecal levels of SCFAs in Control, HF, and SHF groups.

	HF (n=33)	SHF (n=29)	Control (n=15)	*P*
Acetic acid	1286.28 ± 661.61	1303.31 ± 653.97	1301.65 ± 556.98	0.970
Propanoic acid	763.30 ± 494.90	646.18 ± 395.50	637.59 ± 306.94	0.715
Isobutyric acid	92.05 (53.80, 158.67)	71.57 (27.85, 130.96)	104.43 (63.78, 132.84)	0.453
Butyric acid	764.32 ± 689.10	624.64 ± 451.59	502.65 ± 328.88	0.689
Isovaleric acid	108.56 (45.30, 187.76)	68.41 (26.25, 121.89)	102.50 (59.15, 133.87)	0.336
Valeric acid	116.89 (43.19, 198.58)	88.64 (14.02, 193.55)	101.00 (19.04, 154.88)	0.516
Caproic acid	4.04 (1.62, 24.29)	2.86 (0.66, 7.70)	2.94 (1.40, 5.37)	0.244

### 3.6 Relationship between the gut microbiota and clinical parameters with SCFAs

**Figure 5:**
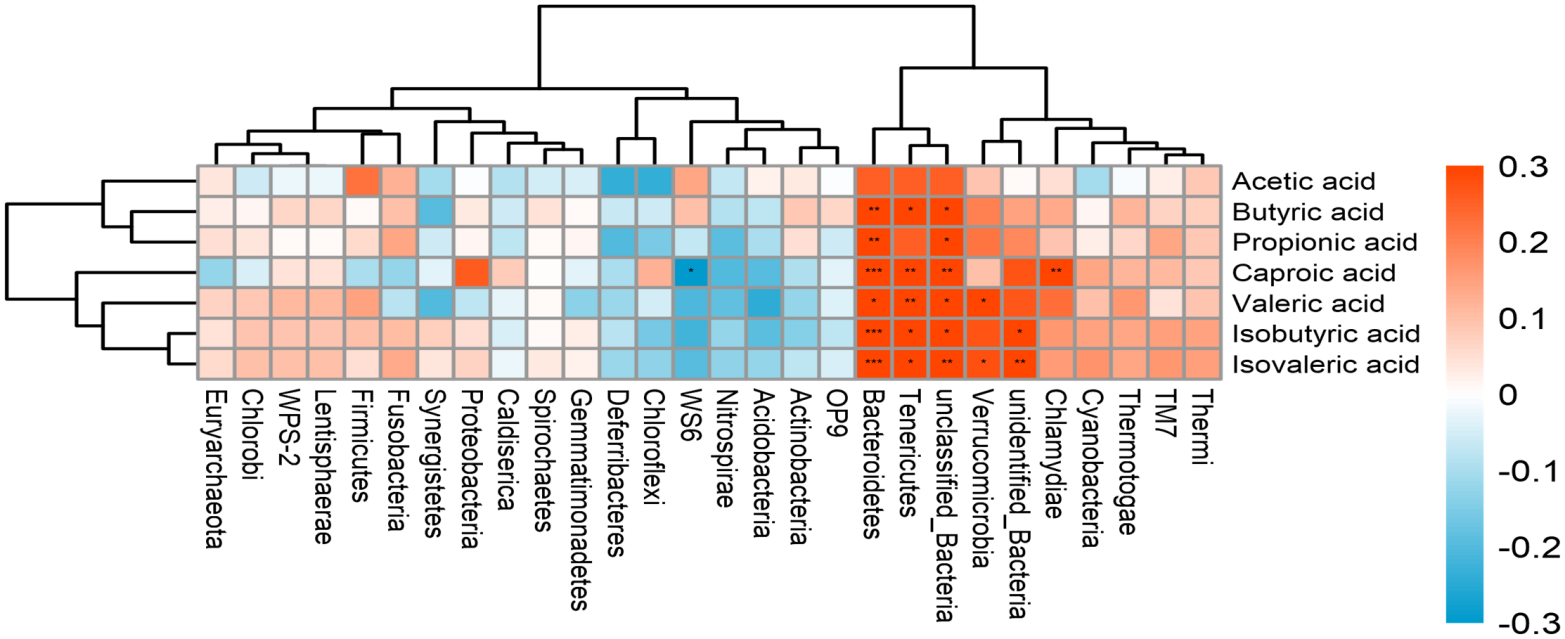
The correlation between the gut microbiota and SCFAs. The intensity of the colors represents the degree of association between the variables measured and asterisks indicate significant associations. *p < 0.05; **p < 0.01, ***p < 0.001.

**Figure 6:**
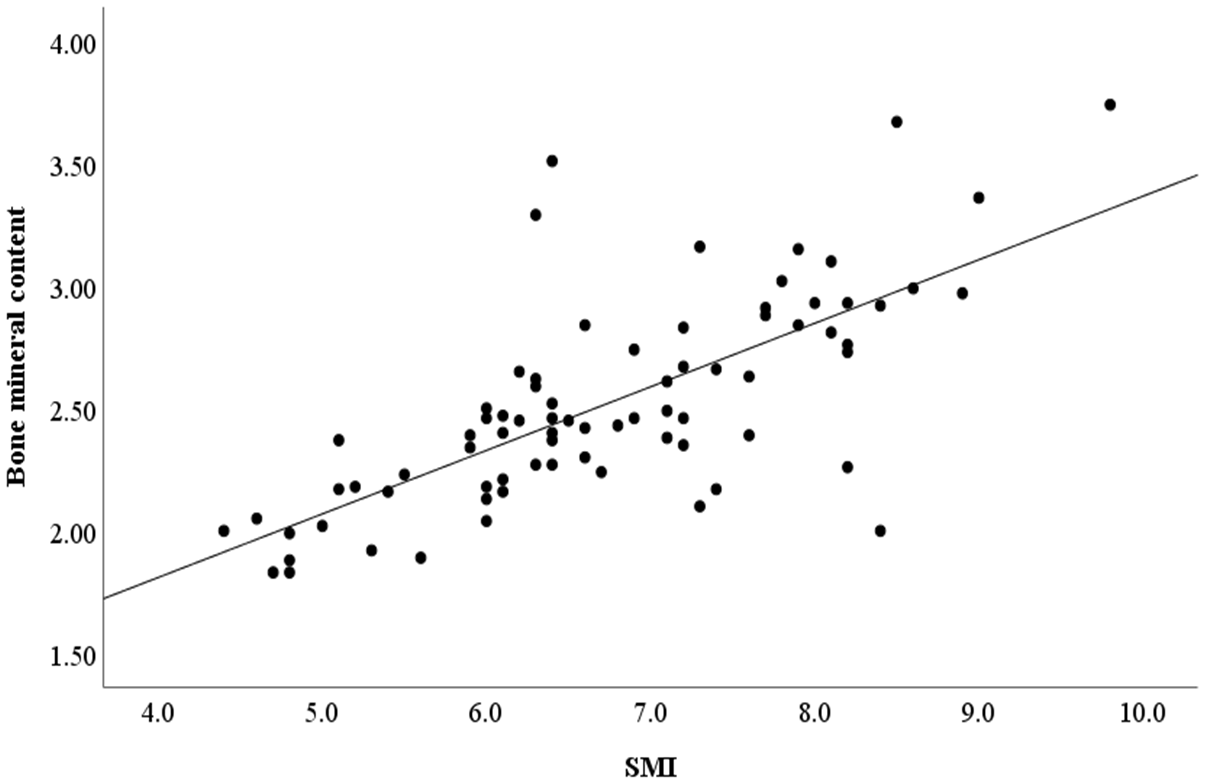
The correlation between SMI and bone mineral content. SMI, skeletal muscle index.

An association analysis was conducted to examine whether the microbiota is associated with SCFA production in all subjects. The results showed *Tenericutes* and *Bacteroidetes* were positively associated with most of SCFAs production. *Verrucomicrobia* was positively correlated with Valeric acid and Isovaleric acid. The abundance of *Chlamydiae* was positively associated with caproic acid (**Figure 5**). In addition, a positive correlation was found between SMI and bone mineral content (**Figure 6**) (r = 0.709, *p*<0.001).

## 4 Discussion

In this study, we first presented the composition of gut microbiota, SCFA levels, and their relationship in HF patients with and without sarcopenia and controls. We found that overall microbial diversity (alpha‐diversity) and community structure (beta‐diversity) were significantly different between the control individuals and HF patients with or without sarcopenia, while no difference between the HF patients with sarcopenia and HF patients without sarcopenia. The *Nocardiaceae*, *Pseudonocardiaceae*, *Leuconostocaceae*, *Alphaproteobacteria*, and *Slackia* were found to be more abundant in HF individuals without sarcopenia. Conversely, the *Synergistetes* showed a higher abundance in HF patients with sarcopenia. In addition, the levels of isobutyric acid, isovaleric acid, and valeric acid were lower in HF patients with sarcopenia compared with that in the HF patients without sarcopenia and control group but has no significant differences.

Microbiota diversity has been proposed as a health biomarker. Diversity analyses indicated a difference in gut microbiota composition between the control group and HF patients with or without sarcopenia in the current study. Loss of gut flora biodiversity is associated with HF patients with and without sarcopenia. Based on a metagenomics study of patients with sarcopenia (Ticinesi et al., 2020), we expected that alpha-diversity and beta-diversity would be reduced in HF patients with sarcopenia compared to HF patients without sarcopenia, but the results exhibited that there were no significant differences between these two groups in our study population.

The main bacterial phyla in the gut are *Firmicutes*, *Bacteroidetes*, *Actinobacteria*, and *Proteobacteria*, which constitute the vast majority of the dominant human intestinal flora (Arumugam et al., 2011). The results were the same in our study. Some studies have examined gut microbiome differences in HF patients compared to control individuals (Sun et al., 2021). Likewise, we found significant differences in the composition of gut microbiome between control and HF patients with or without sarcopenia. In line with the study by Elena et al (Gutierrez-Calabres et al., 2020), *Firmicutes* and *Bacteroidetes* were the two most abundant phyla in control individuals. *Proteobacteria* are gram-negative bacteria, with the outer membrane mainly composed of lipopolysaccharide (LPS). The majority of the *Proteobacteria* phylum are pathogenic bacteria, it is thought that they serve as a microbial signature of dysbiosis in gut microbiota (Shin et al., 2015). An elevated level of *Actinobacteria* in cardiovascular diseases has been reported (Khan et al., 2022). Consistent with these findings, the relative abundance of *Proteobacteria* and *Actinobacteria* were higher in HF patients with and without sarcopenia than that in control individuals.

Differential abundances of certain bacterial taxa were observed in HF patients without sarcopenia compared with the control. Specifically, in line with previous studies, the abundance of *Nocardiaceae* which is associated with steroid hormone synthesis (Sherman et al., 2018), was found to be increased in HF patients without sarcopenia in our study. *Pseudonocardiaceae, Slackia*, and *Alphaproteobacteria* belong to the phylum *Actinobacteria* and *Proteobacteria*, respectively. *Proteobacteria* and *Actinobacteria* have an impact on the prognosis of patients with HF by influencing the production of trimethylamine-N-oxide (Bin-Jumah et al., 2021). The increased abundance of *Proteobacteria* and *Actinobacteria* was also discovered in HF patients without sarcopenia in our study. Based on LEfSe analyses, *Synergistetes* were found abundant in the HF patients with sarcopenia. *Synergistetes* was correlated with infection and enriched in HF patients with preserved ejection fraction (Huang et al., 2021; Zhu et al., 2022). Increasing evidence demonstrated that *Synergistetes* is associated with periodontal diseases, which is prevalent in sarcopenia patients (Belibasakis et al., 2013; Hatta and Ikebe, 2021). It may be a biomarker of HF patients with sarcopenia that influences the progression of sarcopenia in HF patients. *Clostridia*, *Faecalibacterium, Peptostreptococcaceae*, and *Prevotellaceae* were believed to be probiotic species that enriched the fecal microbiomes of control.

Changes in the microbiota of those suffering from various diseases have been associated with reduced diversity of bacteria and the amount of SCFAs in the feces (Machiels et al., 2014; Wang et al., 2014). SCFAs are produced by the gut microbiota metabolizing dietary fiber and undigested carbohydrates. Alterations in fecal SCFAs may lead to dysbiosis of the intestinal microbiota and inflammatory changes. Muscle mass and strength were increased in germ-free mice fed acetate, propionate, and butyrate (Lahiri et al., 2019). SCFAs, particularly butyric acid, can improve muscle atrophy during the aging process (E Walsh et al. (2015)). Our data suggested that there were no significant differences in SCFAs in fecal samples among the three groups. This was in contrast with our hypothesis. Treatment-related confounders in the HF patients with and without sarcopenia cannot be entirely ruled out.

Correlation analysis showed a positive correlation between SCFAs and SCFA-producing bacteria. Different SCFAs are known to be produced by various bacteria. For example, *Bacteroides spp.* produced acetate and propionate (Koh et al., 2016). The abundances of the *Verrucomicrobia* were increased in mice feeding with the butyrate and SCFA mix water (Lee et al., 2022). Metabolism and function of skeletal muscle are susceptible to changes in the gut microbiota, this association appears to be partially mediated by the generation of SCFAs. SCFAs have been proven to influence carbohydrate, lipid, and protein metabolism in skeletal muscle tissues both *in vitro* and *in vivo* (Frampton et al., 2020). Meanwhile, SCFAs inhibit systemic inflammation by binding to G-protein receptors on the surface of the cells and enhance myocardial energy metabolism, which plays a cardioprotective role (Borchers and Pieler, 2010). Moreover, a study on older Koreans found a positive correlation between sarcopenia and bone mineral content (Kim et al., 2014). Lower bone mass may have effects on falls and fracture risk in sarcopenia patients (Gandham et al., 2021). In our study, bone mineral content was positively correlated with SMI. HF patients with sarcopenia had also significantly lower bone mineral content compared with HF patients without sarcopenia.

There are some limitations in our study, including the small sample size, and the lack of detailed information about dietary intake and physical activity. Although all participants were from the same region and had similar dietary habits, we were still unable to fully rule out the influence of diet on gut microbiota and SCFAs. Furthermore, this is a single-center clinical study and may have a selection bias. In addition, a cross-sectional research is difficult to determine the causal relationships. Larger cohort studies are needed to explore the effect of gut flora on sarcopenia in HF patients and whether modulating the gut microbiota and metabolites can alter the natural course of sarcopenia in HF patients.

## 5 Conclusion

In summary, the preliminary study suggests that HF patients with and without sarcopenia differ from control subjects in intestinal microbial composition. *Nocardiaceae*, *Pseudonocardiaceae*, and the *Alphaproteobacteria*, *Slackia* were significantly enriched in HF patients without sarcopenia. *Synergistetes* may be biomarkers of HF patients with sarcopenia. Modulating the gut microbiota may be a new target for the prevention and treatment of sarcopenia in heart failure patients.

## Data availability statement

The original contributions presented in the study are included in the article/supplementary material. Further inquiries can contact the corresponding author. The data presented in the study are deposited in the NCBI repository, accession number PRJNA930609.

## Ethics statement

The study involving human participants was reviewed and approved by Ethics Committee of the Second Xiangya Hospital of Central South University. The patients/participants provided their written informed consent to participate in this study.

## Author contributions

JP: Writing-Original Draft, Investigation, Methodology. HG: Visualization, Methodology. XL: Project administration, Funding acquisition. YL: Software, Date Curation. SL: Conceptualization, Validation. ST: Funding acquisition, Software. LD: Resources, Supervision. XZ: Conceptualization, Funding acquisition, Writing-Reviewing, and Editing. All authors contributed to the article and approved the submitted version.
